# A System of Practical Surgery

**Published:** 1853-06

**Authors:** 


*A System of Practical Surgery.* By WILLIAM FERGUSSON, F. R. S., Professor of Surgery in King's College, London; Surgeon to H. R. H. Prince Albert. Fourth American edition. With three hundred and ninety-three illustrations. Philadelphia: Blanchard & Lea. 1853.

"Good wine needs no blush;" and books of this quality would find a ready sale with a less elaborate and less pictorial sign. "Three hundred and ninety-three illustrations!" At least four times as many as embellish the lintels of any beer-shop in the old town of Mayence: and we think no one will deny that they are good pictures, albeit they are not always very handsome.

"Fergusson's Surgery" has very few faults: as few, perhaps, as any other book of like pretensions. It does not claim to be a complete treatise upon surgery, which would properly embrace nearly all the branches of professional knowledge—no such book has ever yet been published, nor are we certain that it would be desirable. Nor is it, on the other hand, a mere compound or abridgment of surgery—we wish there were fewer of these. It is precisely what it purports to be, a volume on regeonal and operative surgery, almost exclusively: and it is, therefore, eminently practical and useful.

The American edition before us is a republication of the last and enlarged English edition; and we cannot refrain from expressing our obligations to the publishers that they have again saved us the infliction of an American "annotator." We receive it as evidence of an improved taste, and as a favorable omen for the future. The same house ventured, two years ago, to publish, without notes, "Skey" and "Malgaigne." Have the sales proved less

---

remunerative than the sales of "Miller" and "Pirrie," which were issued in 1852, under the auspices of American editors? Gentlemen, look to the figures, and let us know the footing. F. H. H.

